# Use of ELISA to predict high *Mycobacterium avium* subsp. *paratuberculosis* shedding in faeces of infected animals

**DOI:** 10.2478/jvetres-2026-0012

**Published:** 2026-03-31

**Authors:** Věra Fichtelová, Alena Králová, Vladimír Babák, Kamil Kovařčík

**Affiliations:** Department of Infectious Diseases and Preventive Medicine, Department of Microbiology and Antimicrobial Resistance, Veterinary Research Institute, 621 00, Brno, Czech Republic; Department of Microbiology and Antimicrobial Resistance, Veterinary Research Institute, 621 00 Brno, Czech Republic

**Keywords:** faecal samples, Johne’s disease, MAP shedding, paratuberculosis, qPCR

## Abstract

**Introduction:**

Paratuberculosis is a chronic wasting disease caused by *Mycobacterium avium* subsp. *paratuberculosis* (MAP). Control in herds relies on detecting infected animals, which are managed based on the level of MAP shedding. Faecal samples from seropositive animals are examined by bacterial culture and/or qPCR to detect MAP. The aim of this study was to evaluate the use of serology to predict the extent of MAP shedding.

**Material and Methods:**

A total of 139 serum and stool samples were tested. The sample-to-positive ratio (S/P) of the ELISA assay and the crossing point (Cp) of the qPCR test were used to estimate the relative amount of antibodies in serum and MAP in stool, respectively. Spearman’s correlation was used to assess the relationship between S/P and Cp values. Further analyses tested the significance of differences in S/P ratios between groups of animals categorised by their level of MAP shedding.

**Results:**

A weak-to-moderate correlation was found between S/P and Cp values (rs = –0.3471). Significant differences in S/P ratios were identified between groups of animals representing moderate-to-high and low MAP shedding. High MAP shedding was associated with an S/P ≥ 2.5.

**Conclusion:**

Our findings indicate that the ELISA test can be used to predict high MAP shedding. Despite the weak-to-moderate correlation between S/P and MAP levels in faeces, animals with an S/P ≥ 2.5 should be considered high shedders and removed from the herd without delay.

## Introduction

Paratuberculosis (Johne’s disease) is a chronic wasting disease of ruminants caused by infection with *Mycobacterium avium* subsp. *paratuberculosis* (MAP). The infection leads to the development of granulomatous enteritis, causing reduced nutrient and water intake, diarrhoea and eventual death from exhaustion. The negative effects on the profitability of infected cattle herds are primarily a reduction in milk production, growth retardation and early culling.

Infection follows a four-stage course: latent, subclinical, clinical and advanced. Animals are primarily infected orally *via* MAP excreted in colostrum, milk and faeces. In the gastrointestinal tract, MAPs are translocated from the intestinal lumen into enteric tissues, where they are engulfed by resident submucosal macrophages. Inside macrophages, MAP subverts their immune response, preventing the recognition and killing of the internalised bacterium, thereby allowing its replication (1, 11). The progression of the infection is characterised by an increased number of macrophages, proliferating MAP and infected macrophages, and later by the formation of granulomas (8, 9). The spread of infection in the early disease stages is controlled by a cellular immune response. However, in the clinical and advanced stages, a non-protective humoral immune response predominates, with the production of antibodies (Ab) (16). The latent stage of infection is characterised by the absence of MAP and non-detection of Ab. In the subclinical stage of infection, the increasing number of bacteria in the intestinal tissues leads to the shedding of MAP in the faeces. This stage, with intermittent MAP shedding, can last from 2 to 10 years and is responsible for the lengthy incubation period of the disease. In the clinical and advanced stages of infection, significantly higher amounts of macrophages, MAP and infected macrophages are present in the intestinal tissues, accompanied by high levels of MAP shedding in the faeces and elevated Ab titres detected in the serum (16).

To control paratuberculosis in herds, infected animals should be identified and removed to prevent transmission to susceptible cattle. Both direct and indirect methods are commonly used to identify infected animals. The presence of pathogenic bacteria in faeces is determined by bacterial culture and/or by PCR, while the immune response against MAP is evaluated by detection of MAP-specific Ab by ELISA. Infected animals are culled immediately or the culling could be delayed depending on the amount of faecal MAP shedding and the prevalence of infection in the herd; however, culling of animals that shed high amounts of MAP is always necessary (3). Knowledge of the amount of MAP shed in faeces is therefore important for making the decision to cull a particular cow. Bacterial culture results expressed as CFU classify animals as high (≥100 CFU/tube), medium (20 ≤ CFU/tube < 100) or low (<20 CFU/tube) shedders of MAP in their faeces (19). In a qPCR, the amount of MAP is usually estimated semi-quantitatively by the cycle value at which the fluorescence of the tested sample rises above the background fluorescence (threshold cycle (Ct) or crossing point (Cp). This value is inversely proportional to the amount of bacterial DNA put into the reaction. Navarro-Gonzalez *et al*. (19) in their study predicted low faecal shedding with Ct ≥ 31.12, moderate shedding with 31.12 > Ct ≥ 27.23 and high shedding with Ct < 27.23. In addition, other authors have shown an association between the amount of Ab determined by ELISA and the presence of MAP in faeces detected by qPCR and/or faecal culture. (2, 17, 20).

The easy-to-perform ELISA is a time-saving and inexpensive method that is often used in paratuberculosis control programmes as a herd screening tool or to detect infected animals in MAP-positive herds. Serum ELISA is the primary method for detecting infected animals in Czech cattle herds, where cows are tested between six and two weeks before calving. (6). Animals with positive antibody results can then undergo faecal testing by qPCR or culture to detect MAP shedding and substantiate the farmer’s decision to cull.

The aims of our study were to evaluate the correlation between serum Ab levels and MAP stool counts, to assess whether the extent of MAP shedding could be predicted serologically without the need for laborious detection of MAP in stool samples, and to consider the potential use of serum Ab levels in paratuberculosis control programmes.

## Material and Methods

### Blood and faecal samples

Animals were selected based on previous positive ELISA results from blood samples collected as part of the paratuberculosis control programme conducted between 2018 and 2023. In herds positive for paratuberculosis, cows aged two years and older were examined to identify those infected. Owners of antibody-positive cows were asked to re-sample their animals. In total, 139 individual faecal and 139 serum samples were collected. The samples were collected simultaneously, transported to the laboratory, and either processed immediately or stored overnight at 4°C for processing the next day.

### ELISA testing

Blood samples were allowed to clot, and the serum was screened for Ab against MAP using the PTB Ab ELISA 480 (TestLine, Brno, Czech Republic). The relative amount of Ab was calculated by dividing the optical density (OD) of the sample by the OD of the positive control (sample-to-positive ratio, S/P). The results were classified according to the manufacturer’s instructions as positive (S/P > 0.7), negative (S/P < 0.6) or doubtful (S/P = 0.6–0.7). To confirm the presence of specific Ab, each positive serum sample was retested using the ID Screen Paratuberculosis Indirect Screening Test (IDvet, Grabels, France). Only serum samples positive in both tests were used in our study.

### Quantitative PCR (qPCR) testing

Faecal samples were processed, and a qPCR was performed as described previously (7). Extraction of DNA was undertaken from 220 mg of faecal samples with a NucleoSpin DNA kit for genomic DNA (Macherey-Nagel, Düren, Germany) following the manufacturer’s instructions but with two modifications: (1) NucleoSpin Tube A was replaced with a 2-mL screw-cap tube prefilled with 350 mg of 0.1 mm zirconia-silica beads, and (2) samples were homogenised using a MagNaLyser (Roche, Mannheim, Germany) at 7,000 rpm for 60 s.

The qPCR was performed using Luna Universal probe qPCR Master mix (New England BioLabs, Ipswich, MA, USA). A reaction mixture containing ISMav2-F forward primer, ISMav2-R reverse primer and pISMav2-VF hydrolysis probe was prepared according to the manufacturer’s instructions, with a volume of 8 μL of the master mix and 2 μL of the sample DNA. The assay was carried out in a LightCycler 480 Multiwell Plate 96 (Roche) using a LightCycler 480 instrument (Roche), following the manufacturer’s thermocycling conditions. Fluorescence signals were measured at the end of each extension step. Each measurement was performed in triplicate. An internal amplification control was used in a separate reaction tube to monitor the presence of substances inhibitory to qPCR in DNA extractions.

### Faecal culture

Faecal samples were processed and cultured according to the World Organisation for Animal Health Terrestrial Manual (23) with minor modifications: 1 g of faeces was transferred to a 50 mL test tube, shaken with 30 mL of distilled water for 30 min and allowed to stand for 30 min at room temperature. The top 5 mL of the supernatant was then mixed with 25 mL of 0.9% hexadecylpyridinium chloride, shaken for another 30 min and allowed to stand for 72 h in the dark at room temperature. A 200 μL aliquot of sediment was inoculated onto three slants of Herrold’s egg yolk medium with mycobactin. The slants were incubated at 37°C, and bacterial growth was monitored weekly for at least four months. Colonies of MAP were counted, or when the number of colonies could not be it was recorded as uncountable. The identity of bacterial colonies was identified by the qPCR described above.

### Statistical analysis

The normality of all six datasets (*i.e*., S/P values in the CFU < 100 and CFU ≥ 100 groups, S/P values in the Cp < 31 and Cp ≥ 31 groups, as well as Cp values in the CFU < 100 and CFU ≥ 100 groups) was verified using the Shapiro–Wilk test. For two datasets (S/P values in the CFU ≥ 100 group and Cp values in the CFU < 100 group), the null hypothesis that the data follow a normal distribution was rejected (P < 0.01; Shapiro–Wilk test); for the remaining four datasets, the null hypothesis was not rejected (P > 0.05; Shapiro–Wilk test).

Therefore, when comparing the S/P datasets of the CFU < 100 and CFU ≥ 100 groups, as well as the Cp datasets of the CFU < 100 and CFU ≥ 100 groups, a non-parametric test (Mann–Whitney) was used. In the comparison of the S/P datasets of the Cp < 31 and Cp ≥ 31 groups, an unpaired Student’s *t*-test was applied. To assess the relationship between S/P and Cp values, the Spearman’s correlation coefficient was used.

## Results

### Blood and faecal samples

Of the 139 stool samples from ELISA-positive animals, 17 were qPCR negative, of which 7 were culture positive. The mean S/P ratio of serum ELISA of these animals was 1.49. A total of 122 serum and faecal samples with positive results in both ELISA and qPCR were included in the statistical analysis in this study.

### Semiquantitative assessment of MAP faecal shedding by qPCR

The qPCR Cp values of the 122 stool samples ranged from 23.6 to 39.34. Of these, 90 samples had Cp ≥ 31, 22 samples had Cps ranging between 27 and 31 and the remaining 10 samples had Cp < 27. No PCR inhibition was detected in the extracted DNA from any stool sample tested, as demonstrated by the internal control’s successful amplification.

### Faecal culture

A total of 111 faecal samples were examined by bacterial culture. Eleven stool samples were not culture tested because there was not enough material. Growth of MAP was detected in 83 samples, 15 were negative for the presence of MAP colonies and 13 could not be evaluated because of an overgrowth of contaminating microflora. The samples were categorised into groups of <20 CFU/tube, 20–99 CFU/tube and ≥100 CFU/tube, representing low, moderate and high faecal shedding. Small colonies in indistinguishable amounts not possible to count were detected in 27 faecal samples, which is equal to the number of samples with CFU ≥ 100/tube. Moderate CFU counts (20 ≤ CFU/tube < 100) and low CFU counts (CFU/tube < 20) were detected in 8 and 48 stool samples, respectively.

### Correlation of Cp and S/P

To evaluate the relationship between S/P and Cp values, Spearman’s Scorrelation was used. The calculated Spearman’s correlation coefficient was rs = –0.3471 with a 95% confidence interval from –0.4983to –0.1751, indicating a weak-to-moderate correlation between S/P and Cp values. The negative and statistically significant coefficient r_s_ suggests that S/P values tended to decrease with increasing Cp values.

### Evaluation of differences between samples divided according to their Cp value

**Fig. 1.**
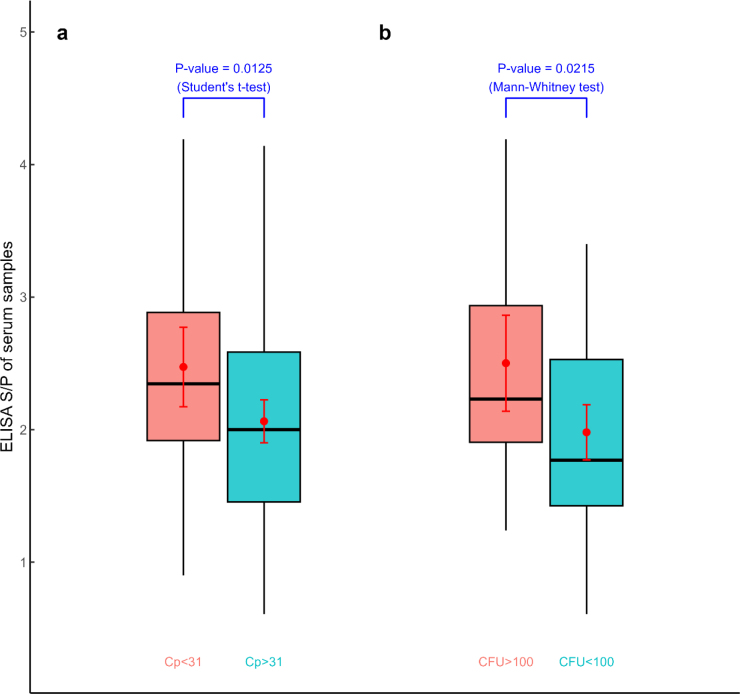
ELISA serum-to-positive (S/P) values of serum samples. (a) Animals grouped according to their qPCR crossing point (Cp) values, representing moderate-to-high (Cp < 31) and low (Cp ≥ 31) *Mycobacterium avium* subsp. *paratuberculosis* (MAP) faecal shedding. (b) Animals grouped according to their CFU count, representing high (CFU ≥ 100) and low (CFU < 100) MAP faecal shedding. Red dots and red line segments within the box plots indicate the group means of the S/P values and the 95% confidence intervals of these means

Groups for which low and moderate (Cp ≥ 27) and high (Cp < 27) MAP faecal shedding were indicated showed mean S/P values of 2.14 and 2.39, respectively. No significant difference was found between groups (P-value = 0.6025). A significant difference (P-value = 0.0125) was found between the mean S/P values of 2.47 for moderate-to-high-shedder samples with Cp values above 31 and 2.06 for low-shedder samples with Cp values below this number (Fig. 1, Table 1).

**Table 1.** Results of Student’s *t*-test for the significance of differences between sample-to-positive ratios (S/P) in ELISA among animals with moderate-to-high (qPCR crossing point (Cp < 31) and low (Cp ≥ 31) *Mycobacterium avium* subsp. *paratuberculosis* faecal shedding

	Cp > 27	Cp < 27	P-value
Mean S/P	2.14	2.39	0.6025
Number of animals	107	15	
	Cp ≥ 31	Cp < 31	
Mean S/P	2.06	2.47	0.0125
Number of animals	90	32	

### Evaluation of differences between groups of animals divided according to their CFU count

**Fig. 2.**
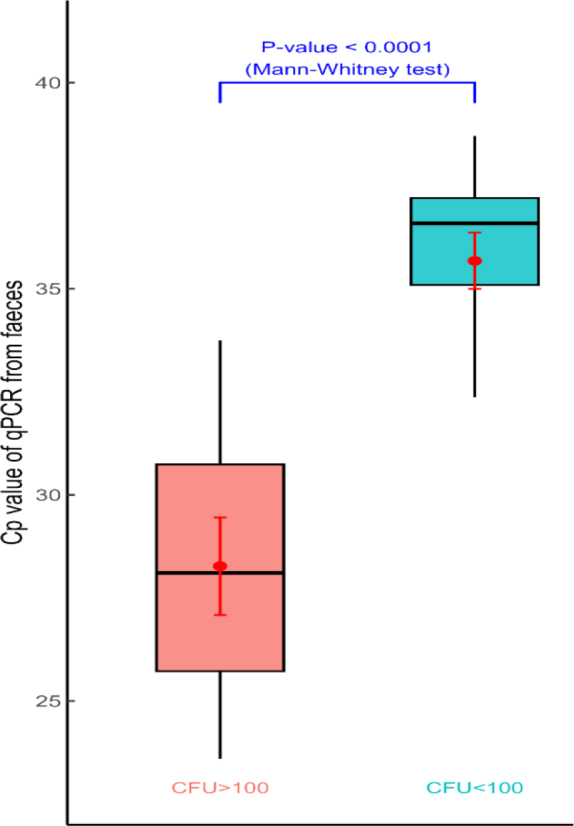
Faecal sample crossing point (Cp) values from qPCR. Samples grouped according to their CFU count, representing high (CFU ≥ 100) and low (CFU < 100) *Mycobacterium avium* subsp. *paratuberculosis* faecal shedding. Red dots and red line segments within the box plots indicate the group means of the Cp values and the 95% confidence intervals of these means

A significant difference (P-value 0.0215) was revealed between S/P values for animals with high (mean S/P 2.5) and low-to-moderate (mean S/P 1.98) MAP faecal shedding (Fig. 1, Table 2). A significant difference (P-value = 0.0001) was also revealed between the mean Cp values of animals belonging to distinct MAP shedding groups (Fig. 2, Table 2).

**Table 2.** Results of Mann–Whitney U test for the significance of differences between sample-to-positive ratios (S/P) in ELISA and crossing points (Cp) from qPCR of samples with lower (CFU < 100) and higher (CFU ≥ 100) *Mycobacterium avium* subsp. *paratuberculosis* faecal shedding

	CFU < 100	CFU ≥ 100	P-value
Mean S/P	1.98	2.5	0.0215
Mean Cp	35.68	28.27	<0.0001
Number of animals	56	27	

## Discussion

A weak-to-moderate non-linear relationship was observed between Cp and S/P values in stool and blood samples from infected animals. The S/P value increased with decreasing Cp value. The Cp value is inversely proportional to the amount of MAP in stool; a lower Cp indicates higher MAP faecal shedding. Thus, our results indicate a weak-to-moderate correlation between serum Ab levels and the amount of MAP in stool samples.

*Mycobacterium avium* subsp. *paratuberculosis* replicates in intestinal macrophages. After the bursting capacity or lifespan of the host cell is reached, the bacteria are released into the extracellular space, where they can remain, be engulfed by macrophages and initiate a new replication cycle, disseminate to another part of the intestine, migrate with lymph to a draining lymph node or be shed into the intestine. Entry to the intestinal lumen is gained as free bacteria through fluid flow or by the migration of macrophages or dendritic cells infected with MAP (11). Shedding of the bacteria begins at the subclinical stage of infection and is associated with MAP proliferation in intestinal tissue. The amount of MAP present in intestinal tissue is influenced by factors related to its replication, such as the replication rate, macrophage lifespan, monocyte recruitment to the site of infection and the expressed immune response. Cell-mediated immunity (CMI) is associated with paucibacillary lesions, whereas antibody-mediated immunity (AMI) correlates with multibacillary lesions (13). Antibody production begins later in the course of infection, after the onset of MAP shedding, and becomes dominant in the clinical stage. It is assumed that Ab production is stimulated by free bacteria accumulated in the intestinal tissue (13, 14).

Longitudinal studies describing the relationship between diagnostic tests detecting specific Ab and MAP faecal shedding have been published previously. In the study by Schukken *et al*. (20), a strong correlation was observed between CFU counts and serum ELISA OD, particularly in samples from animals shedding large amounts of MAP with CFU counts close to or exceeding 100. These animals were termed progressors and showed increasing CFU counts in stool samples and serum Ab over time. These results are consistent with findings by Beaver *et al*. (2), who observed a strong association between milk ELISA OD, faecal qPCR and faecal culture and found that animals categorised as disease progressors gave samples with higher ELISA OD levels. Concordantly with the higher Ab levels in animals with high faecal MAP shedding, animals with sample CFU counts above 100 had a statistically significantly higher ELISA S/P ratio than those with lower MAP shedding. On the other hand, we observed only a low-to-moderate correlation between these two variables, and animals with high serum S/P ratios but low faecal MAP shedding and vice-versa were detected. We assume that the low strength of the correlation may reflect the heterogeneity of the response to MAP infection among infected animals; *i.e*. that the higher number of animals with positive results in both diagnostic tests included in our study did not only include progressors. In addition to animals with progressively increasing Ab levels and sample CFU counts, animals with long-term low Ab production and low MAP shedding, and animals exhibiting a proliferation in the antibody-mediated immune response accompanied by unpredictable low or high sample CFU counts were detected (14). It is generally accepted that CMI is protective, whereas AMI is not. The activity of CMI reduces the number of MAP bacilli in intestinal tissue and is associated with low CFU counts in faeces. Differences in the coordination of the immune response have been proposed, and three distinct interactions between CMI and AMI have been predicted in mathematical modelling to explain the observed variation in MAP shedding patterns and Ab production levels: no interaction, suppression of CMI by AMI and cross suppression (14). The extents of MAP shedding and Ab production are, therefore, influenced by the bacterial load in intestinal tissue and by the efficiency with which cell-mediated and Ab-mediated immune responses interact with each other. The weaker correlation between the level of MAP shedding in faeces and blood Ab values observed in our study among infected animals may reflect the inclusion of cows exhibiting different immune interactions than those proposed for progressors; specifically, the replacement of the originally dominant CMI by Ab production, which subsequently suppresses CMI.

To control infection in herds, transmission of infectious bacteria to susceptible animals should be prevented (4, 22). Calves are the most susceptible to infection because of the presence of ileal Peyer’s patches, which provide a greater number of microfold cells for MAP entry from the intestinal lumen into the intestinal tissue. These transient Peyer’s patches involute during the first year of life, increasing the resistance to infection. Animals become infected orally by sucking colostrum and milk or by swallowing environmental bacteria. The main source of these is infected cows shedding large amounts of MAP in their faeces. Critical steps in controlling paratuberculosis in herds include early removal of calves from contaminated environments to reduce their exposure to adult faeces and the identification and removal of MAP-shedding dams. However, identifying which dams to remove is challenging, because faecal MAP shedding begins at the subclinical stage of infection and precedes Ab production. Animals shed MAP either continuously or intermittently, with periods of shedding interrupted by periods without it. Initial intermittent shedding of small amounts of MAP during the subclinical stage of infection can change to heavy shedding reaching 10^4^ CFU per gram of faeces in super-shedding cows during the clinical and advanced stages of the disease (15). Animals that shed large amounts of bacteria are predominantly continuous shedders and repeatedly positive in ELISA tests (19). In infected herds, a noted distribution of individuals across different stages of the disease is characteristic of a chronic infection, with the herd having a small fraction of clinically ill animals in the most advanced stages (clinical and advanced) and the majority in the subclinical stage (15). Although their numbers are low, cows with high MAP shedding represent a high risk of transmitting the infection and should always be removed from herds.

Use is frequently made of ELISA and PCR to detect animals that should be removed from the herd for effective paratuberculosis control. The sensitivity of these methods to detect MAP-infected animals depends on the stage of infection progression and was found to be highest for high-shedding animals, reaching values of 0.78 (0.68–0.86) and 0.84 (0.77–0.90) for ELISA and PCR, respectively (21). Because MAP shedding precedes Ab production, the sensitivity of ELISA is inferior to that of PCR to detect individuals in the early phase of infection shedding low amounts of MAP (18, 21). However, PCR results must be interpreted with awareness of the intermittent nature of MAP shedding in the initial stage of infection and of the possibility of detection of MAP in the faeces of uninfected animals. *Mycobacterium avium* subsp. *paratuberculosis* can transiently move through the digestive tracts of such animals and be detected in their faeces (5, 10, 16). Despite PCR’s superior sensitivity in early infection, ELISA remains the primary screening tool in most programmes for practical reasons. The low cost and ease of the ELISA method encourage its common use in paratuberculosis control programmes to detect infected animals (22). Upon their detection, a test and cull/manage strategy is applied in infected herds: depending on the level of MAP shedding and the prevalence of infection in the herd, dams and their offspring are either removed promptly from the herd or their removal may be delayed (3, 12). Calves born to low MAP-shedding cows and fed colostrum from MAP-negative dams may become replacement heifers. The level of MAP shedding thus influences the fate of infected animals. Understanding the relationship between ELISA results and actual MAP shedding levels facilitates efficient elimination of paratubercular cows. Our study examined this relationship and found a correlation between ELISA S/P and the qPCR Cp. Statistical analysis further revealed significant differences in S/P values between groups of animals with Cp ≥ 31 and groups with Cp < 31, representing low and moderate to high shedders. These findings are consistent with significant differences detected in S/P and Cp values between cows with high CFU counts (≥100) and those with lower CFU counts (<100) in faeces. Together, these results suggest that high MAP shedding is associated with an S/P of ≥2.5, while low shedding is expected with an S/P of ≤2.0.

Bacterial culture and PCR are used to determine the amount of MAP in faeces. The main disadvantage of bacterial culture is the months-long incubation period required for bacterial growth, whereas PCR provides results within one day. However, both methods are labour-intensive and relatively expensive, and the cost of testing may adversely affect farmers’ willingness to have their animals tested. To effectively control paratuberculosis in herds, all cows aged 2 years and older must be tested repeatedly, at least once a year (12). Shedders are identified by MAP being detected in the faecal samples of ELISA-positive animals. The correlation between Ab levels in serum and MAP in faeces allows ELISA to be used not only for detecting infected animals, but also for categorising them according to the extent of MAP shedding. Two S/P cut off values – 2.5 and 2.0 – have been suggested to distinguish high and low shedders. Animals from which faecal samples have an S/P value of ≥2.5 should be removed from the herd without delay. However, given the low-to-moderate correlation between the S/P ratio and the amount of MAP in faeces, it is recommended to examine faecal samples using MAP detection methods from animals with an S/P <2.5 to avoid misdiagnosing individuals with high bacterial shedding and low S/P values.

## Conclusion

We detected a correlation between the S/P ratio of the ELISA test assessing the amount of Ab in serum and the Cp value of qPCR detecting the number of MAP in stool. This knowledge can be applied in paratuberculosis control programmes conducted in herds to evaluate the risk that infected animals pose in transmitting the infection to susceptible animals. An S/P value ≥2.5 has been associated with CFU ≥ 100, and animals reaching this Ab level should be considered high shedders and removed from the herd without delay. However, because S/P and the amount of MAP in faeces only correlate weakly, it is recommended to test stool samples of seropositive animals with lower S/P values.
